# Effects of seed priming treatments on the germination and development of two rapeseed (*Brassica napus* L.) varieties under the co-influence of low temperature and drought

**DOI:** 10.1371/journal.pone.0257236

**Published:** 2021-09-16

**Authors:** Zong He Zhu, Abdul Sami, Qing Qing Xu, Ling Ling Wu, Wen Yin Zheng, Zhi Peng Chen, Xue Zhi Jin, Hong Zhang, Yong Li, Yan Yu, Ke Jin Zhou

**Affiliations:** College of Agronomy, Anhui Agricultural University, Hefei, China; Department of Agronomy, University of Agriculture, Faisalabad, PAKISTAN

## Abstract

The present study was performed to evaluate the effects of seed priming. This was done by soaking the seeds of two rapeseed cultivars, namely, ZY15 (tolerant to low temperature and drought) and HY49 (sensitive to low temperature and drought), for 12 h in varying solutions: distilled water, 138 mg/L salicylic acid (SA), 300 mg/L gibberellic acid (GA), 89.4 mg/L sodium nitroprusside (SNP), 3000 mg/L calcium chloride (CaCl_2_), and 30 mg/L abscisic acid (ABA). Primed and non-primed seeds were left to germinate at 15°C and -0.15 MPa (T_15_W_15_) and at 25°C and 0 MPa (T_25_W_0_), respectively. The results showed that SA, GA, SNP, CaCl_2_, and ABA significantly improved the germination potential (GP), germination rate (GR), germination index (GI), stem fresh weight (SFW), stem dry weight (SDW), root length (RL), stem length (SL), and seed vigor index (SVI) under T_15_W_15_. For ZY15 seeds under T_25_W_0_, GA, SNP, CaCl_2_, and ABA priming reduced the average germination time (96% after 5 days) compared to that of the control (88% after 5 days). For ZY15 seeds under T_15_W_15_, SA, SNP, CaCl_2_, and ABA priming, with respect to the control and water-treated groups, shortened the average germination time (92% after 5 days) compared to that of the control (80% after 5 days). For HY49 seeds under T_25_W_0_, GA, SNP, CaCl_2_, and ABA priming reduced the average germination time (92% after 5 days) compared to that of the control (85% after 5 days). Similarly, for HY49 seeds under T_15_W_15_, GA priming shortened the average germination time (89% after 5 days) compared to that of the control (83% after 5 days). These priming agents increased the net photosynthesis, stomatal conductivity, and transpiration rate of rape seedlings under conditions of low temperature and drought stress, while also decreasing intercellular carbon dioxide (CO_2_) concentrations. Additionally, SA, GA, SNP, CaCl_2_, and ABA increased superoxide dismutase concentrations (SOD) and ascorbic peroxidase (APX) activities of rape seedlings under stress conditions, while decreasing catalase (CAT) and peroxidase (POD) activities in ZY15 seedlings. In HY49, which is sensitive to low temperature and drought, all priming solutions, except for SNP, led to an increase in SOD activity levels and a decrease in CAT activity levels. Overall, SA, GA, SNP, and CaCl_2_ increased the concentrations of indoleacetic acid (IAA), GA, ABA, and cytokinin (CTK) in seedlings under stress conditions. Moreover, compared to SA, CaCl_2_, and ABA, GA (300 mg/L) and SNP (300 mol/L) showed improved priming effects for ZY15 and HY49 under stress conditions.

## Introduction

In nature, plants are continuously subjected to multiple environmental stresses during their various developmental stages. Drought, extreme temperature, and salinity adversely affect plant growth and quality [1–4]. Drought stress is defined as a prolonged water supply shortage, whether atmospheric (below-average precipitation) or stemming from a lack of surface water or groundwater. In addition, drought stress has a complex effect on plant photosynthesis, reducing the photosynthetic rate and inhibiting photoperiod conversion. It also deregulates ion homeostasis and significantly increases the levels of reactive oxygen species (ROS), leading to oxidative damage to electron transformation, photophosphorylation, and dark reaction processes of cellular components [3, 5–7]. At the morphological and molecular levels, the effects of drought are different. Drought significantly disrupts water-use efficiency, root differentiation, leaf dimensions, stomatal movements, shoot length, cell enlargement, the interaction of water and mineral nutrients, and plant yields [8–10]. For example, polyethylene glycol-induced water scarcity in alfalfa (*Medicago sativa*) decreases hypocotyl elongation, dry and fresh weights of shoots and roots, and ability to germinate. In contrast, polyethylene glycol also enhanced the root length [11].

Low temperature is an abiotic environmental factor that affects plant growth, geographical distribution, and crop yields. There are two types of low temperature stressors. One is the damage caused to plants by temperatures just above the freezing point, called chilling stress. The second is damage caused to plants by freezing stress below 0°C. A previous study found that low temperature stress had a significant effect on chlorophyll fluorescence parameters. In wheat seedlings, low temperature stress significantly reduced leaf chlorophyll content, net photosynthetic and transpiration rates, and stomatal conductance; however, it dramatically increased intercellular carbon dioxide (CO_2_) concentrations [6]. Photosynthesis is a source of dry plant matter accumulation and light-energy conversion. When plants suffer from chilling injury, chlorophyll is reduced, and damage to the chloroplast and thylakoid structures is also inflicted [12]. When plants suffer cold damage, the O_2_ (oxygen) utilization rate decreases, and ROS production increases. The plant body regulates the antioxidant enzyme defense system to remove excess ROS to prevent damage. The main protective plant antioxidant enzymes are superoxide dismutase (SOD), peroxidase (POD), catalase (CAT), and ascorbic peroxidase (APX) [13]. At an early stage of stress, excess hydrogen peroxide (H_2_O_2_) can be removed from the plant body to protect plant cells. However, later stages of stress can accelerate ROS production, reduce chlorophyll content, and promote membrane lipid peroxidation, which, to a certain extent, also occurs as the plant ages [14].

Drought and low temperatures are the most significant threats to global food production, climate change, and global population development. In modern crop production management, seed priming is a promising technique for biotic and abiotic management, which has been known for many years to improve seed germination and the efficacy of stress responses [15, 16]. This technique is a pre-sowing application that permits seeds to germinate more efficiently. It includes hormone priming, hydro priming, chemical priming, halo priming, nutrient priming, and osmo priming [17, 18]. In many horticultural and field crops, improved seed priming techniques are known to decrease emergence time, achieve uniform emergence, and provide better crop standing [19]. Seed priming boosts the synthesis of free radical-scavenging enzymes and increases SOD and CAT expression. Priming with pre-optimized concentrations of priming reagents, such as thiourea, ascorbic acid, salicylic acid, H_2_O_2_, gibberellic acid, and sodium chloride (NaCl), induced metabolic changes leading to improved germination and decreased germination time. There was also a regulatory effect on sunflower seedling growth [20–22].

In wheat under drought stress, CaCl_2_ priming improved seedling establishment, as well as the number of tillers, plant height, grain weight, and grain number [23]. Seed priming with KNO_3_ and urea improved root length, production of proline, germination quality, seedling growth, and protein content in maize hybrids subjected to drought and salinity stress [22, 24]. Hydro priming significantly increases the germination rate during unfavorable conditions of temperature and drought stress in plants [25]. In maize, chitosan, SA, and nitric oxide (NO) priming improved plant biomass, germination rate, and seedling growth under low temperature stress [26, 27]. In cotton and rapeseed, it was observed that a variety of priming agents, such as SA, ABA, GA, jasmonic acid (JA), ethylene, potassium nitrate (KNO_3_), monopotassium phosphate (KH_2_PO_4_), polyethylene glycol (PEG-6000), mannitol, and NaCl, enhanced the tolerance to abiotic stresses (Pb, drought, and temperature) [28, 29].

Brassicaceae is a large family containing several species [30], which include crop hybrids and varieties. Rapeseed is an economically important crop in China and other temperate regions. However, low temperatures and drought stress drastically reduce crop yields. Therefore, reliable methods are required to mitigate the effects of abiotic stress and reduced crop productivity. The current study discusses how seed priming agents (SA, GA, SNP, CaCl_2_, and ABA) induce resistance in rapeseed seedlings to low temperatures and drought stress. Among the five priming agents, which agents perform well? Furthermore, how do they affect the two different varieties of rapeseed? This study provides physiological, molecular, and biochemical parameters to further explore the regulatory mechanisms involved in the low temperature and drought tolerance of rapeseed, mediated by seed priming.

## Test materials and methods

### Seed priming

The experiments were conducted at the laboratory of rapeseed cultivation, College of Agronomy, Anhui Agricultural University in Hefei, China. The seeds of two rapeseed cultivars, Za You 15 (ZY15, registered in China in 2018) and Hui You 49 (HY49, registered in China in 2020), were obtained from Anhui Agricultural University. ZY15 is considered to have resistance to low temperature and drought conditions, whereas HY49 is susceptible to these stresses (unpublished data). Seeds were germinated under two levels of water potential (w): 0.0 MPa (control) and -0.15 MPa, as well as two temperatures, 25°C (control) and 15°C. The drought level was maintained using polyethylene glycol (PEG 6000) [31], which was prepared by dissolving PEG in deionized water (135 g/L) at 15°C. Distilled water, SA (138 mg/L), GA (300 mg/L), SNP (89.4 mg/L), CaCl_2_ (3000 mg/L), and ABA (30 mg/L) were used as priming solutions (the concentrations of the priming agents were selected after a preliminary experiment) and were added to germination boxes along with four layers of filter paper (12 cm × 12 cm × 6 cm). Rape seeds were uniformly placed on the moistened filter paper, sealed within the boxes, and subjected to 25°C and 0 MPa, and to 15°C and -0.15 MPa, for 12 h in the dark. Subsequently, the residual priming solution on the surface of the seeds was immediately rinsed with pure water. The excess water was removed from the seeds with absorbent paper, and the seeds were air-dried to obtain the original weight of the seeds, under natural conditions [32, 33].

### Seed germination and seedling development after priming

After priming, 100 seeds were uniformly placed in a germination box (12 cm × 12 cm × 6 cm) with four layers of filter paper. A germination box containing distilled water was placed at 25°C and 0 MPa (T_25_W_0_) in an artificial climate room, and a germination box containing PEG-6000 solution was placed at 15°C and -0.15 Mpa (T_15_W_15_) in another artificial climate room under a 16/8 h light/dark cycle. Each treatment was repeated three times and the number of germinated seeds was documented daily. After germination, the germination potential (GP), germination rate (GR), mean germination time (GT), germination index (GI), and seed vigor index (SVI) were determined to calculate root and bud length, and the dry weight and fresh weight of the stems (SDW and SFW). The seedlings were then transplanted into nutrient soil for single-cup and single-plant cultivation. The seedlings were grown either at -0.15 MPa, with PEG-6000 in pH 5.5 Hoagland nutrient solution at 15°C, or with only the nutrient solution at 25°C in artificial climate chambers. They received the same volume of water per day. At the end of the germination experiment, the above indices were calculated as follows:

Germination potential (GP) is calculated by the percentage of germinated seeds of the total number of seeds when the germinated seed per day reaches the peak [34].

$\mathrm{GP}\;(\%)=\frac{n_{5d}}{n_{T}}\times 100$, where n_5d_ is the number of germinated seeds at day 5 after sowing and n_T_ is the total number of seeds.

Germination rate (GR) is an estimate of the viability of a population of seeds, which is calculated by dividing the number of germinated seeds on the total number of seeds [35].

$\mathrm{GR}\;(\%)=\frac{n}{n_{T}}\times 100$, where n is the number of germinated seeds and n_T_ is the total number of seeds.

The mean germination time (GT) is calculated to assess the speed of germination [36].

$\mathrm{GT}=\sum (\frac{ni\times di}{n})$, where n represents the number of seeds germinated at day *i*, *d* is the GT in days, and n is the total number of germinated seeds.

The seed germination Index (GI) is considered as the statistical data of the mean germination rate of seeds [37].

GI = ∑(Gt/Dt), which indicates the germination speed. In this equation Gt is the number of germinated seeds and Dt is the corresponding GT.

The seed vigor is the sum of the properties of the seed that determine the potential level of activity and performance of the seed during germination and seedling emergence [38]. The seed vigor index (SVI) is used to assess the seed vigor [37].

SVI = S × GI, where S is the average SFW during the germination period and GI is germination index.

### Measurement of chlorophyll, photosynthetic index, and chlorophyll fluorescence parameters (Fv/Fm)

Chlorophyll, photosynthetic index, and chlorophyll fluorescence parameters (Fv/Fm) were evaluated as previously described [14, 33, 39]. A TYS-A portable chlorophyll meter (PP Systems, USA) was used for chlorophyll assessment. For the determination of photosynthetic indexes, the photosynthetic gas exchange parameters of the rape leaves (Ci, Pn, Gs, and Tr), with essentially identical growth potentials, were calculated outdoors from 8:00 a.m. to 11:30 a.m. using a CIRAS-3 Portable photosynthetic apparatus (PP Systems, USA). The light intensity was set to 1200 mol m^-2^ s^-1^, and the indoor temperature was set to 20°C. Chlorophyll fluorescence was imaged using an IMAGING-PAM instrument (Maxi, Bay instruments, EASTON, MD, USA).

### Measurement of relative electrical conductivity

The relative electrical conductivity (REC) was calculated as previously described [40]. Several rape leaves of the same size, and in the same section of each plant, were cut (without cutting the main vain). The surface was quickly cleaned with tap water, rinsed with pure water three times, and dried with filter paper. Strips of the same length were measured, and three fresh leaves (approximately 0.1 g) were placed in glass tubes containing 10 mL of deionized water before being stored in the refrigerator. After the leaves had soaked at 20°C for 12 h, the initial conductivity of the distilled solvent, referred to as R1, was measured using a conductivity meter (DDS-307A, Shanghai Yoke Instruments Co Ltd., Shanghai, China). The mixture was heated in a water bath for 30 min, placed on ice, and cooled to 20°C. After shaking, the conductivity of the extract was calculated again, referred to as R_2_. The formula for the estimation of REC is as follows:

$$\mathrm{REC}=\frac{R1}{R2}\times 100\%.$$

### Assessment of the relative water content in leaves

Several rape leaves of the same size were cut from the same section of each sample/treatment plant and placed into an aluminum box of known weight to determine the weight of the leaves. This is referred to as the fresh tissue weight (FW) [41, 42]. The plant tissue was soaked in a beaker of deionized water and stored in the dark. After 6–8 h, water was quickly removed from the leaf surface using filter paper. Thereafter, the samples were wrapped in aluminum foil and placed in an oven at 75°C. The leaves were dried at 75°C for 15 min and the dry weight of the tissue (Wd) was weighed using an electronic balance (AE224, Shanghai, China).

### Assessment of antioxidant enzyme activity

Antioxidant enzymes (APX, POD, CAT, and SOD) were measured according to previous studies [13, 43]. Leaf samples were ground into powder with a mortar and pestle on ice in a homogenizing buffer, and then transferred to 5 mL tubes. Homogeneous samples were centrifuged (Shanghai Yoke Instruments Co Ltd., Shanghai, China) at 12,298 RCF (× *g*) at 4°C for 15 min and the supernatants were used for enzyme analysis. Peroxidase activity was tested using guaiacol (Aladdin, Shanghai, China) in 5 mL tubes. The reaction mixture was prepared with 48 mM potassium phosphate buffer (PPB; pH 6.1) (Aladdin, Shanghai, China), 5% guaiacol (w/v), 0.3% H_2_O_2_ (w/v), and enzyme extract. Moreover, the POD absorbance was measured at 470 nm. For CAT activity, 2.5 mL of 50 mM PPB (pH 7.2) and 1.2 mL of 0.3% H_2_O_2_ (w/v) were added to 1.5 mL of enzyme extract and continuously measured at 240 nm for 3 min. The nitro blue tetrazolium (NBT) photochemical technique was used to analyze SOD activity [44]. Samples (0.1 g) were standardized in a 5 mL extractor buffer consisting of 50 mM PPB (pH 7.8). For the analysis, 3 mL of 50 mM PPB (pH 7.8), 30 mM methionine (Aladdin, Shanghai, China), 1 mM EDTA, 750 mM NBT (Aladdin, Shanghai, China), and 21 mM riboflavin were added to samples and analyzed at 560 nm (for the formation of purple formazan). A homogenization buffer containing 8 mM sodium ascorbate (Aladdin, Shanghai, China) was used to detect APX. In the next step, 2 mL of 50 mM PPB (pH 7.2), 0.3 mL of 17 mM ASA (ascorbic acid), 1.5 mL of 0.5 mM H_2_O_2_, and 0.5 mL of extraction enzyme were added while taking continuous measurements on a spectrophotometer (Xevo TQD, Waters, Milford, MA, USA) for 3 min at 290 nm.

### Assessment of ABA, GA, IAA, and CTK

The ABA content was determined as previously described [13]. Leaf samples were crushed into powder with the addition of liquid nitrogen and then homogenized in methanol. Rivier et al. [45] first identified D6-ABA, which was used as an internal standard in this study. In the presence of the Oasis Max solid phase cartridge, methanol was added to formic acid (5% v/v) to purify the materials. Samples were then loaded onto the chromatography-tandem mass spectrometry system, equipped with a triple quadrupole tandem mass spectrometer, and ultra-performance liquid chromatography (UPLC; X8001S, Shanghai Yoke Instruments, Shanghai, China). The GA content was assessed as previously described [14]. Briefly, samples were crushed into powder with the addition of liquid nitrogen, and methanol (80% v/v, Aladdin, Shanghai, China), were used for the extraction. The resulting mixture was purified by ethyl-ether extraction, solid-phase extraction, and reverse-phase extraction. Samples were quantitatively analyzed using capillary electrophoresis-mass spectrometry (CE-MS).

Samples were collected, wrapped in aluminum foil, and placed into the liquid nitrogen bottle at different times during blast inoculation. The CTK process was performed as described previously, using UPLC tandem mass spectrometry (ACQUITY UPLC System/Quattro Ultima Pt; Waters, Milford, MA, USA) with an ODS column (ACQUITY UPLC BEH C18, 2.1 mm × 100 mm, 1.7 μm, Waters, Milford, MA, USA) [46, 47].

The IAA analysis was performed using high-performance liquid chromatography (HPLC) (DW-LC1620A, Shanghai Yoke Instruments, Shanghai, China) [48]. For loading onto the water, we used a Symmetry column C18 (140 mm × 4 mm, 5 μm), and 15 μL samples were placed into a set 15 μL loop. Acetonitrile was used as the solution of mobile phase: methanol: 0.9% acetic acid (5:45:40, v:v:v). Samples were then eluted from the column at 25°C, with a flow rate of 0.8 mL min^-1^, using the Waters Series 515 pump. A photodiode array detector (Waters 2998 Separations Module, Milford, MA, USA), with an absorbance of 218 nm, was used to measure hormonal peaks.

### Statistical analysis

Data are presented as the mean ± SD (standard deviation). Significant differences among treatments were determined using a three-way analysis of variance (ANOVA) (p < 0.05 according to Duncan’s multiple range tests).

## Results

### Effects of priming on germination characteristics under low temperature and drought conditions

Compared with the control group, under T_25_W_0_ (temperature = 25°C and water potential = 0 MPa) no priming agents were able to increase the GP of ZY15 seeds (Table 1). There was no difference between the control and the treated groups (GA, SNP, and ABA). In contrast, SA treatment decreased the GP of ZY15 seeds by 5.3% compared to that of the control. H_2_O, SA, GA, SNP, and ABA priming under T_25_W_0_ increased the GP of HY49 seeds by 4.0%, 2.0%, 4.2%, 4.1%, and 4.4%, respectively, whereas CaCl_2_ priming reduced the GP of HY49 seeds by 1% compared to that of the control. In contrast, H_2_O, SA, GA, SNP, CaCl_2_, and ABA treatments increased the GP of ZY15 seeds by 29.0%, 8.5%, 30.0%, 41.0%, 36.8%, and 38.1%, respectively, under T_15_W_15_ (temperature = 15°C and water potential = -0.15 MPa). The priming solutions SA, GA, SNP, CaCl_2_, and ABA, increased the GP of HY49 seeds by 57.0%, 142.1%, 321.0%, 225.1%, 221.0%, and 244.1%, respectively, compared to that of the control group (Table 1).

**Table 1:** Effects of different seed priming agents on germination characteristics under low temperature and drought conditions.

Cultivar	Priming	Stress	GP (%)	GR (%)	GT (days)	GI	SVI
ZY15	Control	T_25_W_0_	90.00±0.001a	90.00±0.000a	4.09±0.031h	89.73±3.418c	1.81±0.093d
		T_15_W_15_	79.04±1.701d	79.04±1.701c	4.64±0.025de	43.02±0.463f	0.62±0.095hi
	H_2_O	T_25_W_0_	90.00±0.001a	90.00±0.001a	4.02±0.006ij	97.33±0.764ab	2.29±0.161c
		T_15_W_15_	82.05±2.123cd	82.05±2.123c	4.60±0.056e	50.43±1.623e	0.77±0.100gh
	SA	T_25_W_0_	82.05±2.123cd	90.00±0.001a	4.09±0.012h	90.41±1.048c	2.22±0.201c
		T_15_W_15_	80.64±1.065d	86.17±3.314b	4.51±0.044f	44.57±1.242f	0.87±0.081g
	GA	T_25_W_0_	90.00±0.001a	90.00±0.001a	4.02±0.006ij	96.84±1.242b	2.76±0.189b
		T_15_W_15_	86.17±3.314b	86.17±3.314b	4.65±0.023de	50.34±0.367e	0.92±0.060g
	SNP	T_25_W_0_	90.00±0.001a	90.00±0.001a	4.01±0.006j	98.50±1.041ab	2.37±0.324c
		T_15_W_15_	90.00±0.001a	90.00±0.001a	4.53±0.010f	49.98±1.070e	0.97±0.081g
	CaCl_2_	T_25_W_0_	88.09±3.314ab	90.00±0.001a	4.01±0.015j	98.68±1.481ab	3.13±0.123a
		T_15_W_15_	87.29±4.694ab	87.29±4.694ab	4.52±0.029f	48.51±2.162e	0.83±0.071gh
	ABA	T_25_W_0_	90.00±0.001a	90.00±0.001a	4.01±0.001j	98.94±0.098ab	2.31±0.052c
		T_15_W_15_	90.00±0.001a	90.00±0.001a	4.53±0.035f	49.45±0.376e	0.95±0.156g
HY49	Control	T_25_W_0_	86.17±3.314b	86.17±3.314b	4.08±0.036h	90.33±3.884c	1.48±0.055f
		T_15_W_15_	48.25±0.667h	48.83±0.334g	4.60±0.056e	25.83±1.126j	0.33±0.046k
	H_2_O	T_25_W_0_	90.00±0.001a	90.00±0.001a	4.02±0.006ij	97.61±0.348ab	1.58±0.096ef
		T_15_W_15_	58.54±3.025g	58.74±2.861f	4.79±0.055b	29.20±2.502i	0.38±0.017jk
	SA	T_25_W_0_	88.09±3.314ab	88.09±3.314ab	4.07±0.015hi	92.48±2.019c	1.53±0.103ef
		T_15_W_15_	70.94±0.540ef	73.98±1.677d	4.64±0.062de	42.17±2.517fg	0.61±0.091hij
	GA	T_25_W_0_	90.00±0.001a	90.00±0.001a	4.00±0.001j	100.00±0.001a	1.86±0.087d
		T_15_W_15_	90.00±0.001a	90.00±0.001a	4.38±0.044g	60.83±4.072d	0.94±0.080g
	SNP	T_25_W_0_	90.00±0.001a	90.00±0.001a	4.01±0.010j	98.83±0.764ab	1.67±0.097def
		T_15_W_15_	69.53±2.609f	69.53±2.607e	4.66±0.045cd	39.86±2.333gh	0.51±0.035ijk
	CaCl_2_	T_25_W_0_	85.38±4.178bc	86.17±3.314b	4.01±0.010j	98.22±1.345ab	1.55±0.453ef
		T_15_W_15_	68.69±2.961f	71.58±0.956de	4.71±0.04cd	38.07±1.458h	0.46±0.006ijk
	ABA	T_25_W_0_	90.00±0.001a	90.00±0.000a	4.01±0.001j	98.94±0.098ab	1.75±0.142def
		T_15_W_15_	73.65±2.129e	73.65±2.129d	5.23±0.031a	27.26±0.675ij	0.37±0.017k

Averages of germination potential (GP), germination rate (GR), germination time (GT), germination index (GI), and seed vigor index (SVI) are given. Treatments that do not have the same letters are significantly different (p < 0.05) as determined by Duncan’s multiple range tests. Each point represents the mean of three replicates. The values presented are the mean ± standard deviation (SD). Significant differences among treatments were determined using a three-way analysis of variance (ANOVA).

Under T_25_W_0_, the GR of ZY15 seeds after priming treatment did not differ from that of the untreated seeds. However, compared with the untreated group, the priming agents, water, SA, GA, SNP, CaCl_2_, and ABA, increased the GR of ZY15 seeds by 3.8%, 9.0%, 9.0%, 13.9%, 10.4%, and 13.9%, respectively, under T_15_W_15_ (Table 1).

For the control under T_25_W_0_, GA, SNP, CaCl_2_, and ABA priming shortened the average GT of the ZY15 seeds by 1.7%, 2.0%, 2.0%, and 2%, respectively. GA, SNP, CaCl_2_, and ABA priming under T_25_W_0_ induced 96% germination after 5 days of treatment, whereas the control treatment showed 88% germination. SA priming and control treatments exhibited the same results. However, no noteworthy change was recorded in comparison to the water priming under T_25_W_0_. Compared to the control, which showed 85% germination after 5 days, GA, SNP, CaCl_2_, and ABA priming reduced the average GT (92% after 5 days) of HY49 seeds. However, there was no significant difference with water priming under T_25_W_0_ (Table 1).

Under T_15_W_15_, SA, SNP, CaCl_2_, and ABA priming, with respect to the control and water-treated groups, shortened the average GT of ZY15 seeds (92% after 5 days), among which SA priming was the best treatment. The control group showed 80% germination after 5 days, while the GA and control groups showed the same results under T_15_W_15_. Under T_15_W_15_, the average GT of HY49 seeds with SA, SNP, CaCl_2_, and ABA priming increased compared to that of the control. Only GA priming (89% germination after 5 days), compared to the control (83% after 5 days), presented a shortened average GT for HY49 seeds under T_15_W_15_. In relation to the water treatment, all priming agents (except ABA) decreased the average GT of HY49 seeds under T_15_W_15_.

The GI is an estimate of the time, in days, that it takes to arrive at a certain germination percentage [49]. Compared to the control, GA, SNP, CaCl_2_, and ABA improved the ZY15 seed GI by 7.9%, 9.8%, 10.0%, and 10.3%, respectively, under T_25_W_0_. SA treatment also showed a slight increasing trend; however, water treatment was better than SA and GA priming under T_25_W_0_. In relation to the control group, SA, GA, SNP, CaCl_2_, and ABA increased the GI of HY49 seeds by 2.4%, 10.7%, 9.4%, 8.7%, and 9.5%, respectively. Notably, the GI of HY49 seeds were increased by 100% using the GA priming under T_25_W_0_. Water priming also increased the GI compared to that for the control group. Under T_15_W_15_, SA, GA, SNP, CaCl_2_, and ABA priming enhanced the GI of ZY15 seeds by 3.6%, 17.0%, 16.2%, 12.8%, and 14.9%, respectively. There was no change noted between water priming and GA; however, other priming agents decreased the GI of ZY15 seeds. For the HY49 seeds, in comparison to the control under T_15_W_15_, SA, GA, SNP, CaCl_2_, and ABA increased the GI by 63.3%, 135.3%, 54.3%, 47.4%, and 5.5%, respectively. Moreover, compared to the water priming treatment, GA priming showed an improved effect (Table 1).

The SVI is the sum of the properties of the seed that determine the potential level of its activity and performance during germination, and seedling emergence [49]. Under T_25_W_0_, the SVI of ZY15 seeds, in relation to the control, increased with the use of SA, GA, SNP, CaCl_2_, water, and ABA priming by 22.7%, 52.5%, 30.9%, 72.9%, and 27.6%, respectively. Compared to water, CaCl_2_ resulted in a higher SVI. In relation to the control, SA, GA, SNP, CaCl_2_, and ABA increased the SVI by 3.4%, 25.7%, 12.8%, 4.7%, and 18.2%, respectively, for HY49 seeds. No significant change was recorded in comparison to water priming under T_25_W_0_. Compared to the untreated group, SA, GA, SNP, CaCl_2_, and ABA under T_15_W_15_ significantly improved the SVI of ZY15 seeds by 40.3%, 48.4%, 56.5%, 33.9%, and 53.4%, respectively. Simultaneously, no major difference was noted in comparison to water priming. Compared to the control, the SA, GA, SNP, CaCl_2_, and ABA treatments boosted the SVI of HY49 seeds by 84.8%, 184.8%, 54.5%, 39.4%, and 12.1%, respectively, under T_15_W_15_ (Table 1).

### Effects of priming on the biomass of rape seedlings under low temperature and drought conditions

Under T_25_W_0_, all five priming solutions, SA, GA, SNP, CaCl_2_, and ABA, increased the SFW of ZY15 seedlings by 25%, 40%, 20%, 60%, and 15% respectively, compared to that of the control group (Table 2). Moreover, priming with GA and CaCl_2_ significantly increased the SFW, in comparison to water priming, with CaCl_2_ priming producing the best results under T_25_W_0_. The SFW of HY49 seedlings under T_25_W_0_ increased slightly with GA and ABA priming agents, but it was not significantly different when compared to that of the control group. No differences were observed with SA, SNP, and CaCl_2_ priming when compared to that of the control. SA, GA, SNP, CaCl_2_, and ABA (under T_15_W_15_) increased the SFW of ZY15 seedlings by 35.7%, 28.6%, 35.7%, 21.4%, and 35.7%, respectively, compared to that of the control group. In addition, they all also increased the SFW when compared to that of the water priming treatment. In contrast, only GA priming increased the SFW of HY49 seedlings (19.23%), while the results of other priming agents and that of the control were the same under T_15_W_15_.

**Table 2:** Effects of different seed priming strategies on plant biomass under low temperature and drought conditions.

Cultivar	Priming	Stress	SFW (g)	SDW (g)	RL (cm)	SL (cm)
ZY15	Control	T_25_W_0_	0.20±0.004d	0.03±0.002cde	4.77±0.551d	1.17±0.058f-i
		T_15_W_15_	0.14±0.021j-n	0.02±0.002ij	1.83±0.058gh	0.87±0.058k
	H_2_O	T_25_W_0_	0.24±0.018c	0.04±0.002b-e	5.10±0.200cd	1.20±0.173e-i
		T_15_W_15_	0.15±0.024i-m	0.03±0.001c-f	2.03±0.416gh	1.03±0.231ijk
	SA	T_25_W_0_	0.25±0.020c	0.04±0.002ab	5.37±0.723a-d	1.57±0.569bc
		T_15_W_15_	0.19±0.013de	0.03±0.001cde	2.43±0.404fg	1.03±0.058ijk
	GA	T_25_W_0_	0.28±0.022b	0.03±0.003b-e	5.87±0.252abc	1.97±0.115a
		T_15_W_15_	0.18±0.010d-h	0.03±0.004c-g	2.03±0.208gh	1.60±0.100b
	SNP	T_25_W_0_	0.24±0.031c	0.04±0.003bc	5.33±0.586bcd	1.30±0.100d-h
		T_15_W_15_	0.19±0.020de	0.03±0.003b-e	3.77±0.351e	1.13±0.115g-j
	CaCl_2_	T_25_W_0_	0.32±0.011a	0.04±0.003a	5.10±0.346cd	1.20±0.173e-i
		T_15_W_15_	0.17±0.010e-j	0.03±0.004b-e	2.10±0.100gh	0.90±0.100jk
	ABA	T_25_W_0_	0.23±0.005c	0.04±0.001bcd	5.70±0.265abc	1.40±0.100b-f
		T_15_W_15_	0.19±0.030def	0.03±0.001b-e	2.40±0.173g	1.30±0.001d-h
HY49	Control	T_25_W_0_	0.16±0.008g-k	0.03±0.002e-i	5.50±0.700a-d	1.00±0.001ijk
		T_15_W_15_	0.13±0.013mn	0.02±0.001j	1.33±0.058h	0.83±0.058k
	H_2_O	T_25_W_0_	0.16±0.010g-k	0.03±0.002e-h	5.57±0.681abc	1.33±0.058c-g
		T_15_W_15_	0.13±0.007lmn	0.02±0.002j	1.53±0.153h	0.97±0.058ijk
	SA	T_25_W_0_	0.17±0.015f-j	0.03±0.003c-f	6.00±0.361ab	1.37±0.058b-g
		T_15_W_15_	0.14±0.015j-n	0.03±0.001g-j	3.23±0.737e	1.37±0.058b-g
	GA	T_25_W_0_	0.19±0.009d-g	0.03±0.002c-f	5.83±0.351abc	1.90±0.001a
		T_15_W_15_	0.15±0.006h-m	0.03±0.002f-j	3.70±0.600e	1.50±0.001bcd
	SNP	T_25_W_0_	0.17±0.011e-j	0.03±0.003d-g	5.93±1.250ab	1.37±0.058b-g
		T_15_W_15_	0.13±0.012mn	0.03±0.001f-j	1.53±0.153h	1.07±0.058h-k
	CaCl_2_	T_25_W_0_	0.16±0.044g-l	0.03±0.001e-i	5.47±0.551a-d	1.53±0.231bcd
		T_15_W_15_	0.12±0.005n	0.03±0.001g-j	3.63±0.252e	1.13±0.153g-j
	ABA	T_25_W_0_	0.18±0.015d-i	0.03±0.002c-f	6.13±0.153a	1.43±0.058b-e
		T_15_W_15_	0.14±0.008k-n	0.02±0.003hij	3.20±0.200ef	1.37±0.058b-g

Averages of stem fresh weight (SFW), stem dry weight (SDW), root length (RL), and stem length (SL) are given. Treatments that do not have the same letters are significantly different (p < 0.05) as determined by Duncan’s multiple range tests. Each point represents the mean of three replicates. The values presented are the mean ± standard deviation (SD). Significant differences among treatments were determined using a three-way analysis of variance (ANOVA).

Regarding the control, under T_25_W_0_, SA, SNP, CaCl_2_, and ABA slightly increased the SDW of ZY15 seedlings, whereas GA priming decreased the SDW with respect to water priming. In comparison with the untreated group, the five priming solutions, SA, GA, SNP, CaCl_2_, and ABA, increased the SDW of ZY15 seedlings by 50.1%, 50.5%, 50.4%, 50.5%, and 50.8%, respectively, under T_15_W_15_. No major difference was noted when comparing the water priming group (Table 2). After priming with SA, GA, SNP, CaCl_2_, and ABA, the SDW of HY49 seedlings were not considerably different from that of the control, under both T_25_W_0_ and T_15_W_15_.

In comparison to the untreated group under the T_25_W_0_ condition, SA, GA, SNP, CaCl_2_, and ABA priming considerably increased the root length (RL) of ZY15 seedlings by 12.6%, 23.1%, 11.7%, 6.9%, and 19.5%, respectively. Additionally, compared with water priming under T_25_W_0_, all other priming agents, except for CaCl_2_, showed an increasing trend. The four priming solutions, SA, GA, SNP, and ABA, improved the RL in HY49 seedlings under T_25_W_0_ by 9.1%, 6.0%, 7.8%, and 11.5%, respectively. Compared to the control, under T_15_W_15_, SA, GA, SNP, CaCl2, and ABA priming significantly increased the RL of ZY15 seedlings by 32.8%, 10.9%, 10.6%, 14.8%, and 31.1%, respectively. Furthermore, compared to water priming under T_15_W_15_, all other priming agents showed an increasing trend, except for GA. Similarly, under T_15_W_15_, SA, GA, SNP, CaCl_2_, and ABA enhanced the RL of HY49 seedlings by 142.9%, 178.2%, 150.0%, 172.9%, and 140.6%, respectively, compared to that of the control (Table 2).

With regard to the control group under T_25_W_0_ and T_15_W_15_, SA, GA, SNP, CaCl_2_, and ABA priming significantly increased the stem length (SL) of ZY15 seedlings; however, the GA treatment showed the best results. SA and GA also considerably improved the SL when compared to water priming under T_25_W_0_ and T_15_W_15_. Under T_25_W_0_, the SL of HY49 seedlings was increased by all five priming agents (SA, GA, SNP, CaCl_2_, and ABA) by 37%, 90%, 37%, 53%, and 43%, respectively, compared to that of the control group. In comparison to the untreated group (under T_15_W_15_), SA, GA, CaCl_2_, SNP, and ABA improved the SL of HY49 seedlings by 65.1%, 80.7%, 28.9%, 36.1%, and 65.1%, respectively, among which SNP had the best effect (Table 2).

### Effects of priming agents on the photosynthetic characteristics of rape seedlings under low temperature and drought conditions

**Fig 1:**
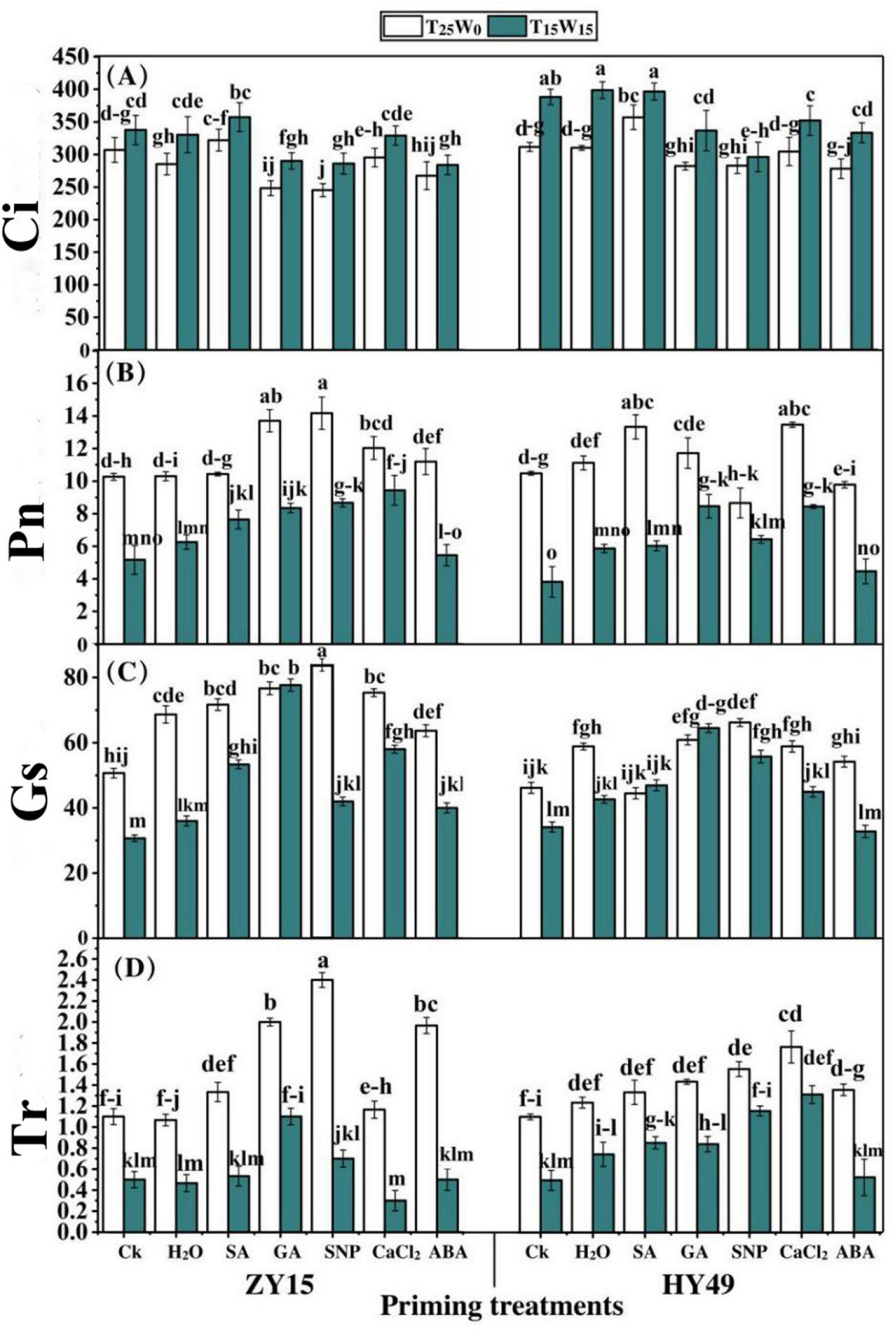
Effects of priming agents on the photosynthetic characteristics of rape seedlings under low temperature and drought conditions. The effects of low temperature and drought stresses (25°C and 0 MPa, 15°C and -0.15 MPa) on the photosynthetic characteristics: (**A**) intercellular carbon dioxide (Ci), (**B**) net photosynthesis (Pn), (**C**) stomatal conductance (Gs), and (**D**) transpiration rate (Tr) of *Brassica napus* seedlings (varieties ZY15 and HY49). Seven different treatments (**a**) control (Ck), (**b**) water (H_2_O), (**c**) salicylic acid (SA), (**d**) gibberellic acid (GA), (**e**) sodium nitroprusside (SNP), (**f**) calcium chloride (CaCl_2_), and (**g**) abscisic acid (ABA) were evaluated. Each point represents the mean of three replicates. The values presented are the mean ± standard deviation (SD). Significant differences among treatments were determined using a three-way analysis of variance (ANOVA) (p < 0.05 according to Duncan’s multiple range tests).

Under T_25_W_0_, GA, SNP, and ABA reduced the intercellular carbon dioxide (Ci) concentration in ZY15 seedling leaves by 19.1%, 20.0%, and 12.9%, respectively, compared to that of the control group (Fig 1A). In comparison, SA priming increased the Ci concentration of ZY15 leaves by 11.6%. Furthermore, with respect to water treatment, GA reduced intercellular carbon by 44.7%. In comparison to the control and water priming treatments, SA priming under T_25_W_0_ improved the Ci concentration of HY49 seedling leaves by 14.7%. Under T_15_W_15_, GA, SNP, and ABA treatments reduced the Ci concentration of ZY15 seedling leaves by 14.1%, 15.3%, and 31.6%, respectively, compared to that of the control and water priming groups. In comparison to the control group, GA, SNP, CaCl_2_, and ABA priming, reduced the Ci concentration (by 13.3%, 23.8%, 9.3%, and 14.2%, respectively) of HY49 seedling leaves under T_15_W_15_ (Fig 1A).

Compared to the control and water priming treatments, GA and SNP priming under T_25_W_0_ significantly enhanced the net photosynthesis (Pn) of ZY15 seedling leaves by 33.4% and 38.0%, respectively (Fig 1B). In contrast, CaCl_2_ and ABA slightly improved the Pn of ZY15 leaves. Compared to the control, SA, GA, and CaCl_2_ increased the Pn of HY49 seedling leaves, under T_25_W_0_, by 29.1%, 26.2%, and 29.3%, respectively, and significantly improved the Pn when compared to that of water priming. In relation to the untreated and water priming groups, SA, GA, SNP, and CaCl_2_ significantly increased the Pn of ZY15 seedling leaves under T_15_W_15_ by 48.0%, 61.5%, 67.7%, and 82.6%, respectively. Compared to the control group, under T_15_W_15_, SA, GA, SNP, and CaCl_2_ increased the Pn of HY49 seedling leaves by 7.4%, 12.6%, 7.6%, and 12.1%, respectively (Fig 1B).

In comparison to the control group, SA, GA, SNP, CaCl_2_, and ABA, enhanced the stomatal conductance (Gs) of ZY15 seedling leaves by 41.4%, 51.3%, 83.6%, 49.3%, and 25.7%, respectively, under T_25_W_0_ (Fig 1C). GA, SNP, and CaCl_2_ also improved the Gs when compared to that of water priming. Furthermore, compared to the control group, under T_25_W_0_, GA, SNP, and CaCl_2_ enhanced the Gs of HY49 seedling leaves by 31.9%, 43.5%, and 27.5%, respectively, whereas no significant difference was recorded with water priming. In relation to the control and water priming groups, SA, GA, SNP, CaCl_2_, and ABA enhanced the Gs of ZY15 seedling leaves under T_15_W_15_ by 73.9%, 153.3%, 33.7%, 89.1%, and 30.4%, respectively. Compared to the control under T_15_W_15_, SA, GA, and SNP increased the Gs of the HY49 seedling leaves by 37.6%, 89.0%, and 63.4%, respectively (Fig 1C).

SA, GA, SNP, and ABA increased the transpiration rate (Tr) of ZY15 seedling leaves by 21.2%, 81.8%, 118.2%, and 78.7%, respectively, under T_25_W_0_, compared to that of the control (Fig 1D). Additionally, the Tr was improved by 41.3% and 60.5% with SNP and CaCl_2_ treatments, respectively, in the HY49 seedling leaves under T_25_W_0_, compared to that of the control. Under T_15_W_15_, GA increased the transpiration rate of ZY15 seedling leaves up to 120.0%, compared to that of the control and water priming treatments. With respect to the control and water priming treatments under T_15_W_15_, SNP and CaCl_2_ increased the transpiration rate of HY49 seedling leaves by 132.9% and 164.3%, respectively.

### Effects of priming on the relative electrical conductivity and relative water content

**Fig 2:**
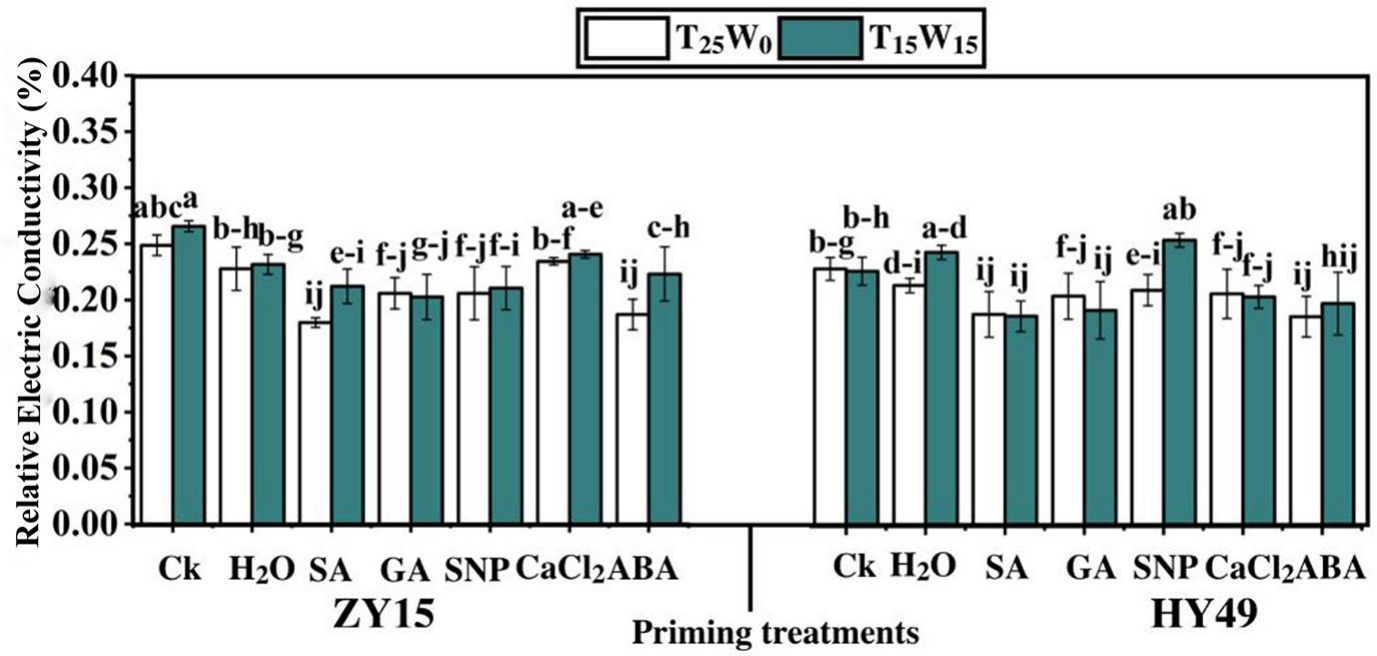
Effects of low temperature and drought stresses (25°C and 0 MPa, 15°C and -0.15 MPa) on the relative electrical conductivity (%) of *Brassica napus* seedlings (varieties ZY15 and HY49). Seven different treatments (**a**) control (Ck), (**b**) water (H_2_O), (**c**) salicylic acid (SA), (**d**) gibberellic acid (GA), (**e**) sodium nitroprusside (SNP), (**f**) calcium chloride (CaCl_2_), and (**g**) abscisic acid (ABA) were evaluated. Each point represents the mean of three replicates. The values presented are the mean ± standard deviation (SD). Significant differences among treatments were determined by a three-way analysis of variance (ANOVA) (p < 0.05 according to Duncan’s multiple range tests).

Under T_25_W_0_, SA, GA, SNP, and ABA decreased the REC of ZY15 seedling leaves by 27.7%, 17.2%, 17.2%, and 24.7%, respectively, compared to that of the control group (Fig 2). Moreover, the RECs under water and CaCl_2_ priming were the same. Compared to the control group, SA, GA, SNP, CaCl_2_, and ABA priming (under T_25_W_0_) decreased the REC of HY49 seedling leaves by 17.6%, 13.7%, 14.3%, 12.9%, and 18.5%, respectively. Under T_15_W_15_, SA, GA, SNP, CaCl_2_, and ABA considerably reduced the REC of ZY15 seedling leaves by 20.1%, 23.8%, 20.7%, and 16.0%, respectively, compared to that of the control group (Fig 2). With respect to the control group, SA, GA, CaCl_2_, and ABA priming reduced the REC of HY49 seedling leaves by 17.6%, 12.7%, 10.5%, and 9.9%, respectively.

**Fig 3:**
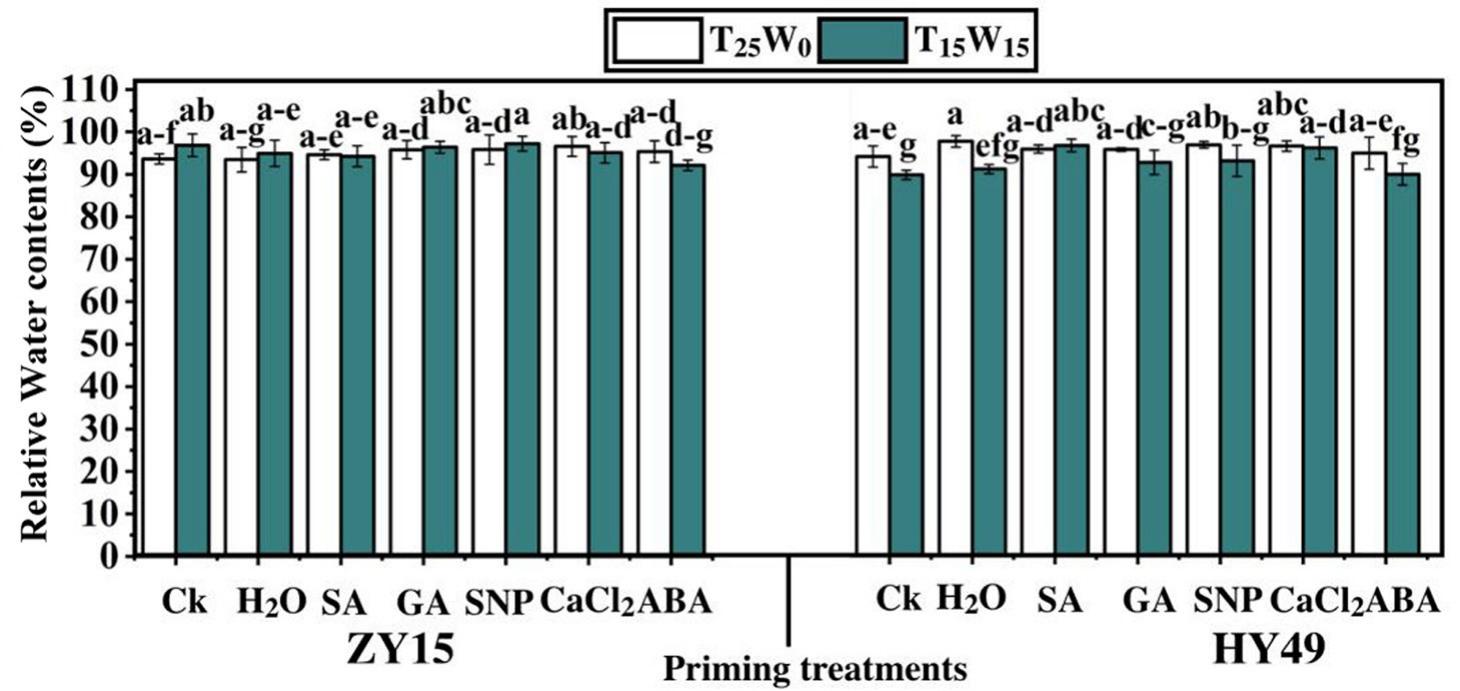
Effects of low temperature and drought stresses (25°C and 0 MPa, 15°C and -0.15 MPa) on the relative water contents of *Brassica napus* seedlings (varieties ZY15 and HY49). Seven different treatments (**a**) control (Ck), (**b**) water (H_2_O), (**c**) salicylic acid (SA), (**d**) gibberellic acid (GA), (**e**) sodium nitroprusside (SNP), (**f**) calcium chloride (CaCl_2_), and (**g**) abscisic acid (ABA) were evaluated. Each point represents the mean of three replicates. The values presented are the mean ± standard deviation (SD). Significant differences among treatments were determined by a three-way analysis of variance (ANOVA) (p < 0.05 according to Duncan’s multiple range tests).

The relative water content of ZY15 and HY49 seedling leaves induced by the five priming agents showed no remarkable difference when compared to that of the control under T_25_W_0_ (Fig 3). However, under T_15_W_15_, the ABA treatment reduced the relative water content of ZY15 leaves at the seedling stage. SA, SNP, and CaCl_2_ slightly increased the relative water content of HY49 seedling leaves under T_15_W_15_ by 7.7%, 7.6%, and 7.1%, respectively, compared to that of the control.

### Effects of priming on antioxidant enzyme activities

**Fig 4:**
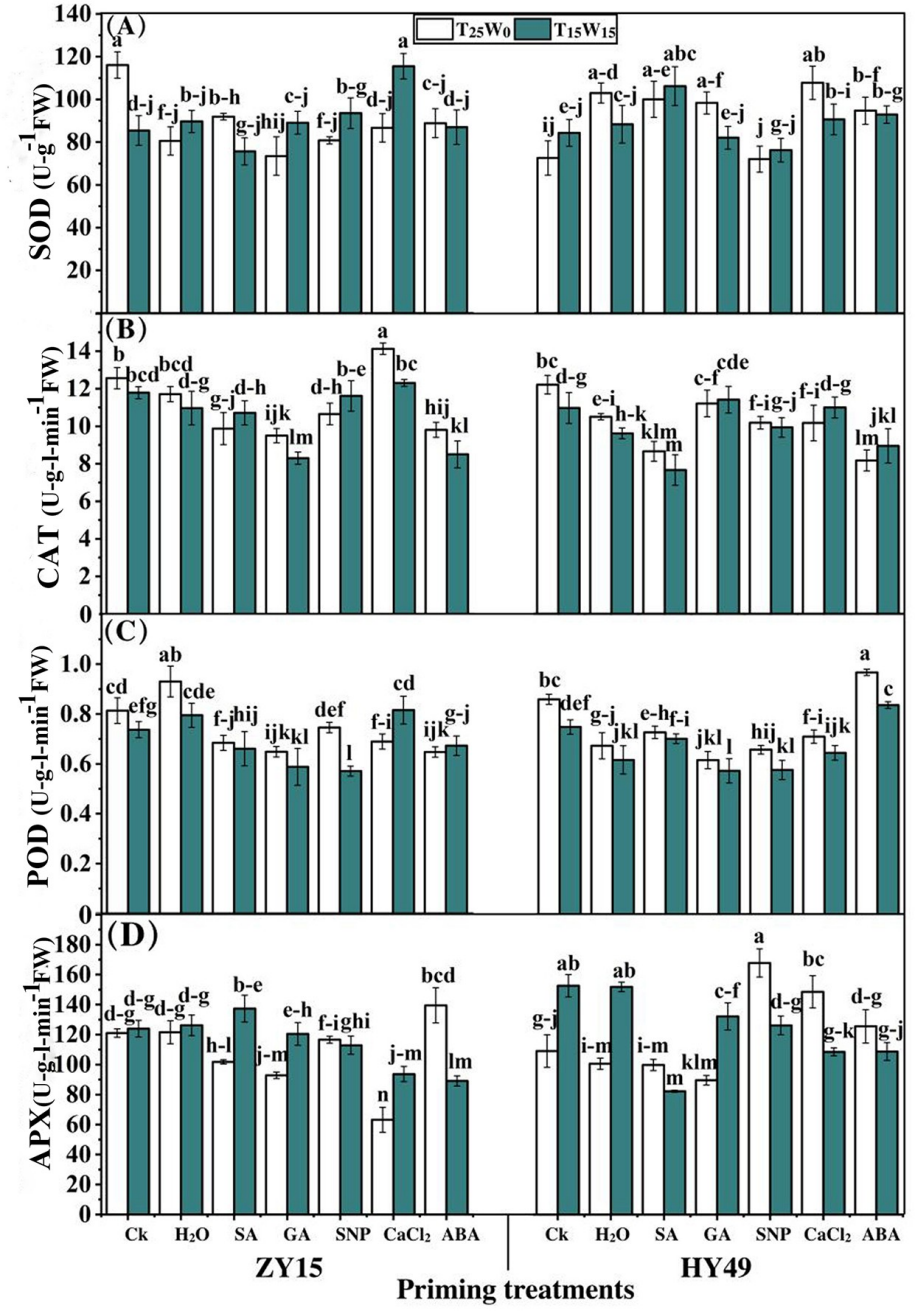
Effects of different priming solutions on antioxidant enzyme activities of rape seedlings under low temperature and drought conditions. Effects of low temperature and drought stressed (25°C and 0 MPa, 15°C and -0.15 MPa) on antioxidant enzyme activity with (**A**) superoxide dismutase (SOD), (**B**) catalase (CAT), (**C**) peroxidase (POD), and (**D**) ascorbate peroxidase (APX) in *Brassica napus* seedlings (varieties ZY15 and HY49). Seven different treatments were evaluated: (**a**) control (Ck), (**b**) water (H_2_O), (**c**) salicylic acid (SA), (**d**) gibberellic acid (GA), (**e**) sodium nitroprusside (SNP), (**f**) calcium chloride (CaCl_2_), and (**g**) abscisic acid (ABA). Each point represents the mean of three replicates. The values presented are the mean ± standard deviation (SD). Significant differences among treatments were determined by a three-way analysis of variance (ANOVA) (p < 0.05 according to Duncan’s multiple range tests).

Compared to the control group, SA, GA, SNP, CaCl_2_, and ABA reduced SOD activity levels in ZY15 seedling leaves under T_25_W_0_ by 20.0%, 32.2%, 26.9%, 23.0%, and 23.6%, respectively (Fig 4A). Compared to the control group, SA, GA, CaCl_2_, and ABA priming under T_25_W_0_ increased SOD activity levels by 40.9%, 49.6%, 51.8%, and 24.0%, respectively, in HY49 seedling leaves. SA, CaCl_2_, and ABA also increased SOD activity level in HY49 seedling leaves, when compared to that of the water treatment, under T_25_W_0_. Notably, SA priming was significantly different from the water treatment for SOD. GA, SNP, and CaCl_2_ under T_15_W_15_ improved SOD activity levels in ZY15 seedling leaves by 2.6%, 3.8%, and 5.4%, respectively, compared to that of the untreated group, while CaCl_2_ priming significantly improved SOD activity levels when compared to those of the control and water priming groups. Similarly, SA, CaCl_2_, and ABA priming under T_15_W_15_ increased SOD activity levels in HY49 seedling leaves by 36.6%, 31.8%, and 28.0%, respectively, compared to that of the control.

The CAT levels under T_25_W_0_ in ZY15 seedling leaves were reduced by 21.4%, 24.4%, 15.2%, and 21.9%, after SA, GA, SNP, and ABA treatments, respectively, compared to that of the control group (Fig 4B). Furthermore, compared to the control group, SA, SNP, CaCl_2_, and ABA reduced CAT activity levels in HY49 seedling leaves by 29.1%, 16.6%, 16.7%, and 33.1%, respectively; of which, SA and ABA priming showed significantly different results from the water treatment under T_25_W_0_. GA and ABA treatment under T_15_W_15_ decreased CAT activity levels in ZY15 seedling leaves by 29.6% and 27.9%, respectively. In HY49 seedling leaves, SA and ABA treatment under T_15_W_15_ reduced CAT activity levels by 30.1% and 18.4%, respectively, compared to that of the control. Additionally, the SA treatment yielded significantly different levels from that of the water treatment.

Under T_25_W_0_, SA, GA, CaCl_2_, and ABA, reduced POD activity levels in ZY15 seedling leaves by 15.9%, 20.3%, 15.3%, and 20.4%, respectively, compared to that of the control (Fig 4C). Meanwhile, under T_25_W_0_, SA, GA, SNP, and CaCl_2_ reduced POD activity levels in HY49 seedling leaves by 15.4%, 28.3%, 23.4%, and 17.4%, respectively, compared to that of the control. Compared to the control, under T_15_W_15_, SA, GA, and SNP decreased POD activity levels in ZY15 seedling leaves by 10.3%, 20.2%, and 22.5%, respectively. SA, GA, and CaCl_2_ reduced POD activity levels under T_15_W_15_ by 23.4%, 22.9%, and 13.8%, respectively, in HY49 seedling leaves. In comparison, SA and ABA increased POD activity levels when compared to that of the water priming treatment.

Under T_25_W_0_, the ABA treatment increased APX activity levels by 25.6% in ZY15 seedling leaves compared to those of the control and water priming groups (Fig 4D). SNP, CaCl_2_, and ABA priming, under T_25_W_0_, considerably increased the APX activity levels of HY49 seedling leaves by 21.3%, 48.1%, and 23.0%, respectively, compared to that of the control. Moreover, compared to the control and water priming treatments, SA priming under T_15_W_15_ improved the APX activity levels of ZY15 seedling leaves by 17.14%. In contrast, none of the other priming agents increased APX activity levels under T_15_W_15_. Compared to the control, SA, GA, SNP, CaCl_2_, and ABA priming reduced the APX activity levels of HY49 seedling leaves under T_15_W_15_ by 35.0%, 19.3%, 24.3%, 29.0%, and 29.0%, respectively.

### Effects of priming on hormones

**Fig 5:**
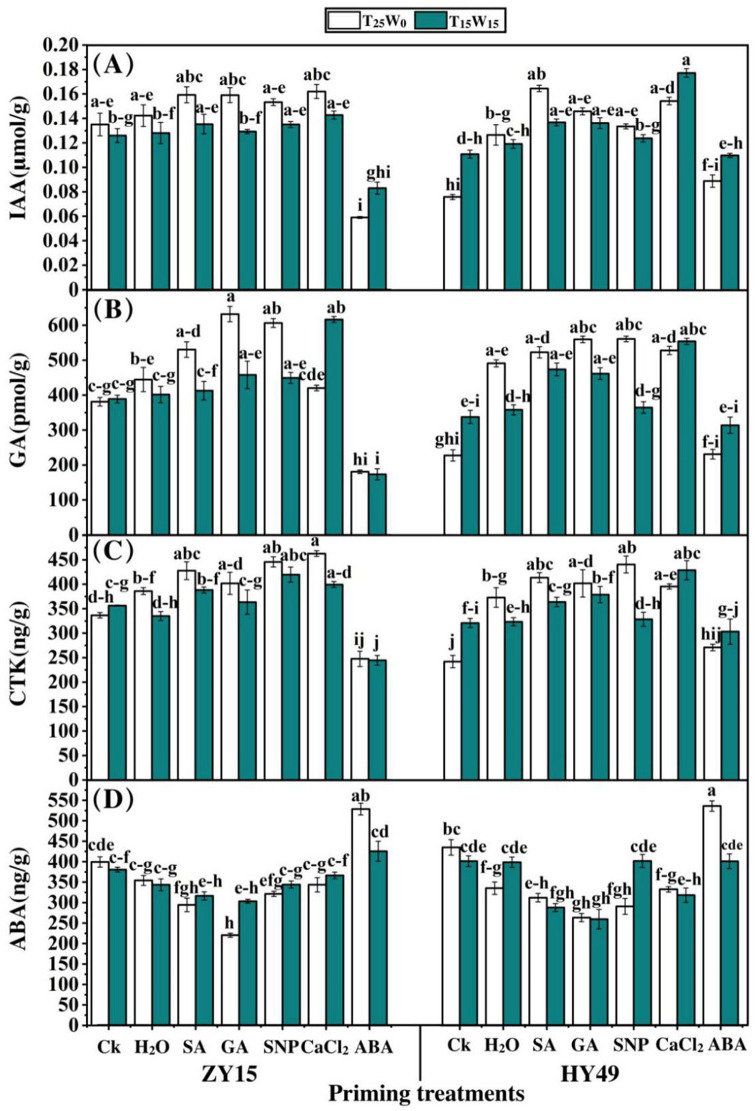
Influence of priming on the hormones of rape seedlings under low temperature and drought conditions. Effects of low temperature and drought stresses (25°C and 0 MPa, 15°C and -0.15 MPa) on the hormones: (**A**) indoleacetic acid (IAA), (**B**) gibberellic acid (GA), (**C**) cytokinin (CTK), and (**D**) abscisic acid (ABA) of *Brassica napus* seedlings (varieties ZY15 and HY49). Seven different treatments, namely, (**a**) control (Ck), (**b**) water (H_2_O), (**c**) salicylic acid (SA), (**d**) gibberellic acid (GA), (**e**) sodium nitroprusside (SNP), (**f**) calcium chloride (CaCl_2_), and (**g**) abscisic acid (ABA), were evaluated. Each point represents the mean of three replicates. The values presented are the mean ± standard deviation (SD). Significant differences among treatments were determined by a three-way analysis of variance (ANOVA) (p < 0.05 according to Duncan’s multiple range tests).

The ABA treatment reduced the IAA level of ZY15 seedling leaves by 56.4% under T_25_W_0_, compared to that of the control and water priming treatments (Fig 5A). In contrast, SA, GA, SNP, and CaCl_2_ increased the IAA levels in ZY15 seedling leaves by 30.2%, 30.3%, 28.2%, and 30.1%, respectively, under T_25_W_0_. In HY49 seedling leaves, under T_25_W_0_, SA, GA, SNP, CaCl_2_, and ABA improved the IAA level by 115.2%, 91.3%, 74.7%, and 102.1%, respectively, compared to that of the control. This study also showed significant differences among the SA, GA, SNP, and water priming treatments. The ABA treatment reduced the IAA level of ZY15 seedling leaves by 34.0% compared to that of the control and water treatments, while the other priming agents increased the IAA level under T_15_W_15_. In contrast, IAA content in leaves primed with SA showed the highest increase compared to that of the control. Under T_15_W_15_, CaCl_2_ improved the IAA content in HY49 seedling leaves by 59.7% compared to that of the control.

Under T_25_W_0_, SA, GA, and SNP priming enhanced the GA levels in ZY15 seedling leaves by 44.3%, 65.8%, and 59.1%, respectively, compared to that of the control (Fig 5B). The ABA treatment significantly reduced the GA levels of ZY15 seedling leaves, under T_25_W_0_, by 52.6% and 59.4%, respectively, compared to those of the control and water treatment groups. In HY49 seedling leaves, SA, GA, SNP, and CaCl_2_, under T_25_W_0_, improved the GA content by 129.3%, 145.4%, 146.0%, and 131.6%, respectively, compared to that of the control. CaCl_2_ priming significantly enhanced the GA content of ZY15 seedling leaves by 63.9% and 54.6%, respectively, compared to those of the untreated and water priming groups under T_15_W_15_. Moreover, under T_15_W_15_, the ABA treatment considerably decreased the GA content of ZY15 seedling leaves relative to the control and water priming treatments by 55.4% and 56.8%, respectively. Under T_15_W_15_, CaCl_2_ priming enhanced the GA content of HY49 seedling leaves by 63.9% compared to that of the control.

SA, GA, SNP, and CaCl_2_ significantly increased the CTK level under T_25_W_0_ in ZY15 seedling leaves by 27.1%, 22.4%, 32.5%, and 37.5%, respectively, compared to that of the control (Fig 5C). The ABA treatment under T_25_W_0_ considerably reduced the CTK levels of ZY15 seedling leaves by 26.4% and 35.8%, respectively, compared to those of the control and water treatments. Under T_25_W_0_, SA, GA, SNP, and CaCl_2_ improved the CTK levels in HY49 seedling leaves by 70.8%, 65.9%, 81.8%, and 63.2%, respectively. Under T_15_W_15_, the ABA treatment reduced the CTK level of ZY15 seedling leaves by 31.4% compared to that of the control. Meanwhile, also under T_15_W_15_, CaCl_2_ increased the CTK level by 33.6%, compared to those of the control and water treatments.

Under T_25_W_0_, SA, GA, SNP, and CaCl_2_ decreased ABA levels in ZY15 seedling leaves by 26.3%, 23.0%, 21.3%, and 44.8%, respectively, compared to that of the control (Fig 5D). However, the ABA level of ZY15 seedling leaves was enhanced by 32.4% compared to that of the control. Similarly, SA, GA, SNP, and CaCl_2_ (under T_25_W_0_) reduced ABA levels in HY49 seedling leaves by 28.2%, 35.9%, 32.2%, and 26.1%, respectively. Under T_25_W_0_, ABA priming increased ABA levels in HY49 seedling leaves by 23.2% and 59.8%, respectively, compared to those of the control and water priming treatments. Furthermore, under T_15_W_15_, SA, GA, SNP, and CaCl_2_ reduced the ABA content in ZY15 leaves by 28.3%, 39.4%, 33.1%, and 23.6%, respectively. SA and GA significantly decreased the ABA level in HY49 seedling leaves by 28.2% and 35.3%, respectively, under T_15_W_15_, compared to that of the control.

## Discussion

Abiotic stresses, such as low or high temperature, insufficient or excessive water, high salinity, heavy metals, and ultraviolet radiation, are harmful for plant growth and development. These stresses result in a considerable amount of agricultural production losses globally [8, 50, 51]. Drought/low temperature stress disrupts the plant’s defense system by altering gene expression, knocking out resistant genes, and disrupting protein/enzyme activities, causing cell injury, cell division, and osmotic stress [51–53]. This type of stress also disrupts stomatal density, causes protein denature, disrupts signaling molecules (such as ABA, H_2_O_2_, and NO), aggravates fatty acids, affects transcriptional control, and disrupts antioxidant enzymes and hormones [8, 53, 54]. The present study suggests priming agents as a means to improve plant defense mechanisms by increasing the expression of stress resistance genes; balancing ionic homeostasis, membrane functions, and transcriptional control by transcription factors; balancing antioxidant activities; inducing the activation of osmoprotectants; and cellular homeostasis. Further studies at molecular level will prove it.

Low temperature stress considerably reduced the GP, GR, and SVI of rapeseed and extended the average GT in different crops [35–37]. In the present study, water priming significantly improved the ability of the two cultivars to germinate and significantly shortened the GT. It has been found that seeds placed under conditions of insufficient water do not retain the minimum amount of water and are, therefore, unable to initiate germination [54, 55]. For example, under the conditions of insufficient water, Kaya et al. [56] found a decrease in final seed germination rate of the conventional sunflower and pepper seeds. These results are consistent with those of the present study [22, 57]. In the present study, five priming solutions significantly increased the germination capability of ZY15 and HY49, promoted stem and root length, and increased the fresh and dry weights of the stems (Tables 1 and 2). However, they had different effects on shortening the GT and improving the GI and SVI (Tables 1 and 2). In the present study, the germination percentage decreased dramatically with declining water potential, due to decreased water uptake [54, 58]. ZY15 showed better performance than HY49 when treated with all five priming agents. As ZY15 already has resistance to low temperature and drought, present study suspect that these priming agents increased its ability to germinate and its resistance to stress. According to Jisha et al. [17], primed seeds can quickly imbibe and restore seed metabolism, thereby increasing the rate of germination. In several crops, seed germination and early seedling growth are the most vulnerable stages for water limitation. Water stress can decrease the amount and uniformity of germination, contributing to poor yield and poor yield efficiency [59, 60]. Notably, a substantial reduction in germination was observed as an effect of CaCl_2_ priming. CaCl_2_ priming improves root development more efficiently under optimal sowing conditions [61, 62].

In general, the fresh and dry stem weights and the root length of the two varieties of seedlings were improved to a certain extent by the five priming solutions, under both optimal and low temperature conditions. Previous studies have shown that, as maize seeds were subjected to cold stress, the dry weight of roots and shoots and the activities of defense enzymes in SA-treated seeds were higher than those in untreated grains [63]. Salicylic acid and hydrogen peroxide have been reported to improve cold resistance and facilitate seed germination in maize. Few studies have focused on the combined impact of SA and H_2_O_2_ on the improvement of cold tolerance in maize; therefore, the process behind this combined effect is still unclear [63]. The present findings suggest that GA and ABA may have great potential as priming agents in rapeseed production, by improving the tolerance to low temperature and drought. Gibberellic acid and water priming greatly improved seedling weight by supplying hydrolyzing products. Research has shown that loss of dry weight by seedlings under drought stress is due to decreased hydrolyzed reserves and is not linked to the output of seed deposits [60, 64]. The key function of GA is to stimulate genes encoding for enzymes involved in seed germination, particularly alpha-amylase, by upregulating mRNA expression [65]. From the perspective of the improvement of all indexes, GA priming had the best effect on improving seed germination and seedling growth (Tables 1 and 2) [65].

The results of the present study are consistent with those of previous studies showing that seed priming solutions significantly increased the photosynthetic conversion rate of seedlings [65, 66]. Drought stress substantially lowers the photosynthetic rate, whereas seed priming agents regulate photosynthetic rate [67, 68]. Previous studies also supported the present results, which show that the physiological processes by which plants react to both salinity and drought are identical. This indicates that both stresses must be interpreted by the plant cell as water deficiency [3, 69]. The Ca^2+^ is an essential mineral nutrient for plant growth and development, and its main role is to maintain cell membrane stability and to regulate the transport of inorganic salt ions [32, 70]. While Ca^2+^ is crucial for controlling plant tolerance to both drought and salinity, the relationship between Ca^2+^ and salinity has been more intensively studied than drought [71]. When water availability is low, the diffusion of carbon dioxide through the stomata decreases. The present results indicate that CaCl_2_ treatment could cause this phenomenon [62, 67].

Seed priming can significantly improve the tolerance of cold and drought stress of different crop varieties in different environments [33, 67]. This study suggests that the treatment of seedlings with appropriate concentrations of priming solutions could improve stress resistance. In maize plants, SA priming increased the biosynthesis of antioxidant enzymes under low temperatures [29, 72, 73]. In addition, the levels of the antioxidant enzymes, SOD and CAT, as well as that of the hormones (except ABA), increased after GA treatment (Figs 4 and 5), which is consistent with the results of previously published results [33, 74]. This may be due to the direct or indirect activation of antioxidant enzymes by GA, which promotes the synthesis of various enzymes. The present study showed that low temperature and drought stress extensively disturbed the plant antioxidant mechanism and hormonal balance, while seed priming agents played a vital role in the repair of these systems. Previous studies have shown that the complex synthesis, and catabolic balance of ABA and GA is essential for seed germination [58, 75]. By depositing osmoprotectants, abscisic acid seed priming serves as a growth regulator in plant systems under restricted soil moisture conditions [76]. Further, crop priming with GA increases the germination of rye seeds and stimulates antioxidant production under drought stress [58, 77].

In addition, CaCl_2_ priming has a significant effect on endogenous hormones and significantly increases the levels of IAA, GA, and CTK [63, 70]. In low temperature and drought conditions, ABA can control bud dormancy in plants by upregulating hormone transcription factors, thereby maintaining normal plant growth [14]. Sodium nitroprusside is often used as a donor for NO, which is a bioactive molecule that plays a crucial role in signal transmission. Studies have shown that exogenous NO can significantly improve seedling quality, while increasing the activity levels of SOD, CAT, and other antioxidants, in addition to the levels of IAA, GA, and CTK hormones. In this way, it improves the resilience of plants [14, 22]. The GA enhances the enzymatic activity of isocitrate lyase in the glyoxylate cycle, and triggers the synthesis of soluble sugars and triacylglycerol degradation [27, 78]. Gibberellic acid priming improved the emergence of *N*. *sativa* under severe stress conditions [7, 78]. Further, CaCl_2_ osmo priming of seeds is useful for the development and growth of cereal crops, including rice [70, 79]. Drought and low temperatures disrupt the mineral-nutrient relationship in plants by disrupting the supply, transport, and partitioning of nutrients.

The priming phase contributes to the membrane repair system, by improving membrane stability, as the primed seed has a lower electrical conductivity level [80]. Related findings were obtained from sorghum seeds primed with PEG 6000 [4, 81]. Plants grown with SA priming exhibited enhanced levels of hydrogen peroxide, electrolyte leakage, superoxide anions, lipid peroxidation, and increased ROS enzyme production [62]. A previous study supports the results presented here, where unprimed control seeds recorded the highest value; whereas, the seeds primed with SA had the lowest recorded value in maize [31]. An increase in electrolyte leakage, which reflects a loss in the ability of biological membranes to regulate the transport of ions, has been reported in soybeans under drought stress [82]. Seeds primed with phenylalanine or calcium nitrate had the lowest electrical conductivity readings, suggesting lower degrees of cell injury [31]. However, the electrical conductivity of seed leachate in salicylic acid did not vary from that of mannitol (2% and 4%), gibberellic acid, and hydro priming.

## Conclusion

Treatment with five priming agents (138 mg/L SA, 300 mg/L GA, 89.4 mg/L SNP, 3000 mg/L CaCl_2_, and 30 mg/L ABA) significantly improved the GP, GR, GI, biomass, antioxidant levels, photosynthetic activity levels, and SVI of the two rapeseed varieties. These priming agents also balanced IAA, GA, CTK, and ABA levels. It was concluded that 300 mg/L of GA and 89.4 mg/L of SNP showed the most significant effects against low temperature and drought conditions in rapeseed. Nevertheless, more studies are required to evaluate why GA and SNP priming were better than the other priming agents. The present study also revealed that among the two varieties, ZY15 showed a better performance than HY49; however, seed priming improved the performance of HY49 compared to that of the control. The performance of ZY15 was the best, perhaps because it was already considered to be a stress-tolerant variety. However, the challenges and opportunities associated with various other priming agents and methods need to be addressed, and a cost-efficient technique is required to overcome the challenges in food security.

## Supporting information

S1 Data Abdul Sami raw data. (ZIP)
